# Analysis on the impact of internet use on residents’ tourism consumption behavior and the mechanism of action

**DOI:** 10.1371/journal.pone.0311998

**Published:** 2024-10-11

**Authors:** Xiankai Lei, Dongmei Yang

**Affiliations:** 1 Business School, Xinyang Normal University, Xinyang, China; 2 Dabie Mountain Economic and Social Development Research Center, Xinyang Normal University, Xinyang, China; 3 School of Marxism, Xinyang Normal University, Xinyang, China; University of Naples Federico II: Universita degli Studi di Napoli Federico II, ITALY

## Abstract

Tourism consumption as a typical representative of service consumption, has strong comprehensiveness and driving force, continuously expanding new consumption upgrade space. In the context of the digital economy, the development of the mobile internet has opened up new opportunities for the tourism industry acting as an important factor influencing the consumer behaviour of the residents. Based on 9007 resident survey data from the China General Social Survey (CGSS) in 2017, this paper explores the relationship between Internet use, access to information and residents’ travel consumption behavior by means of probit model and mediating effect model. First, according to the findings, Internet use has a positive impact on residents’ travel consumption behavior, increasing residents’ tourism expenditure. Second, the mechanism suggests that access to information plays a partially mediating role between Internet use and travel consumption behavior. Third, further analysis revealed that, for the retired population, the more frequently they use the Internet, the more likely they are to spend on travel. Compared to those with lower incomes, those with higher incomes will spend less on travel after retirement. With this in mind, in order to promote residents’ tourism consumption, speed and fee reduction should be continuously promoted, urban and rural information infrastructure should be strengthened, and accessibility of tourism information for residents should be improved. At the same time, there is a need to innovate the way tourism products are promoted, improve the types of tourism products and launch diversified tourism products.

## Introduction

According to statistics from the Ministry of Culture and Tourism, the domestic tourism scale in China increased from 2.957 billion person-times in 2012 to 6.006 billion person-times in 2019. Tourism consumption also rose from 2.27 trillion yuan to 5.73 trillion yuan during the same period. In 2019, the contribution of tourism consumption to China’s GDP reached 11.05%, further driving the country’s economic growth. In the context of the rapid development of internet information technology, the internet use has offered more opportunities for the development of China’s tourism industry. At present, online consumption has become an important way for residents to purchase tourism products and services. Even under the adverse impact of the epidemic, China’s online travel booking user scale still reached 342 million, showing a good development trend. Over recent years, the scale of tourism in China has seen continuous expansion. As such, the development of tourism can not only directly contribute to the development of the national economy and optimize the economic structure, but also promote the transfer of surplus rural labour to employment, increase residents’ income and narrow the income gap between urban and rural areas. According to a survey conducted by the China Tourism Academy, the post-epidemic era has ushered in new growth in China’s tourism industry, with domestic tourism arrivals and tourism revenue exceeding 200 million and 100 billion yuan respectively during 2021 May Day period alone. Overall, however, China’s tourism industry still runs into problems such as a single tourism product, serious homogenization, lack of innovation and difficulty in matching the individual needs of travellers. As one of the "troika”, household consumption is particularly important to the development of the national economy. As a major participant in the tourism industry, residents’ tourism consumption behavior naturally affects the healthy and sustainable development of the industry. Against the above background, it is of great theoretical and practical significance to explore what kind of impact the popularity and use of the Internet has on residents’ tourism consumption behavior and the path of such impact, so as to promote the supply-side structural reform of China’s tourism industry to meet people’s growing demand for tourism consumption.

At present, scholars at home and abroad have explored residents’ tourism consumption behavior from different perspectives, among which, the influencing factors on residents’ tourism consumption behavior can be divided into macro and micro aspects. Macro-level factors cover the economic, social, cultural and ecological environment. The stability of a country or region’s economic environment is conducive to the development of tourism, providing a precondition for residents to travel [1]; climate change, such as global warming, can also contribute to an increase in the probability of residents travelling [2], while the ecological environment of a tourist destination has an important impact on residents’ tourism consumption behavior, with air pollution, such as haze, discouraging residents from travelling [3]. On the back of the rapid development of high-speed railway, the well established high-speed railway transport network influences residents’ tourism consumption behavior by strengthening their consumption perceptions [4]. Algassim (2021) Point out the COVID-19 pandemic influences travel inclination, tourist attitudes and preferences [5].

At the micro level, this includes factors in both family characteristics and individual characteristics. Household economic status is a non-negligible variable, and the travel consumption behavior of household members inevitably takes into account the household’s economic status [6], while the subjective willingness to travel of other household members also has an impact on individual travel consumption behaviour [7]. Individuals’ risk perceptions [8] and particularly, the perceived value of tourists influence their tourism consumption behavior by affecting personal satisfaction [9].Tang (2023) use the PLS-SEM method to elucidate the implementation path and mechanism of green consumption willingness of tourists in urban and rural tourism destinations, it was found that perceived usefulness plays a significant mediating role [10]. In addition, individual characteristics such as age and gender can also influence their travel consumption behaviour [11]. Santos et al (2022) point out emotions and involvement demonstrate greater progress and scientific development to the level of tourism [12]. Ataul (2023) found that green price sensitivity, attitude, subjective norm, and perceived behavioral control positively influence tourists’ intention to visit green hotels in Malaysia [13].

Regarding research related to the Internet and tourism, more literature has explored the impact of the Internet on the tourism industry from a macro level, and the use of Internet to develop the tourism industry is a new breakthrough for the future development of the tourism industry [14]. As a new form of technological diffusion, the Internet can drive the innovative development of tourism and the renewal of tourism products [15], which in turn enhances the competitive advantage of the tourism industry [16]. For different types of tourism businesses, however, the Internet has had a greater impact on travel agents than on tourist hotels [17]. In addition, the Internet can boost national or regional tourism trade, a boost that is more evident in developed countries [18]. In contrast, there is relatively little literature examining the relationship between the Internet and residents’ tourism consumption behaviour at the micro level, with some scholars suggesting that online communication can influence residents’ tourism decisions [19]. Also some scholars point out that Internet use promotes the increase of household tourism consumption to a certain extent [20], but there is no specific analysis of the impact on individual residents or the mechanism of the role of Internet use on residents’ tourism consumption behavior. Literature research has found that compared to the important role of the popularity and use of the Internet in enhancing the quality of tourism consumption and stimulating the vitality of tourism consumption, research on the impact of Internet use on tourism consumption is seriously lagging behind. Accordingly, this paper explores the impact of Internet use on residents’ tourism consumption behavior and the mechanism of action from the perspective of information access, by employing 9007 China General Social Survey (CGSS) data.

The marginal contributions of this paper are, firstly, analyze the impact of the Internet on residents’ tourism consumption from a macro perspective, integrating digital technology and residents’ tourism consumption into a unified research framework. Secondly, from the perspective of information acquisition, exploring the mechanism of the impact of Internet usage on residents’ tourism consumption behavior provides a new explanation for the consumer effects of Internet usage. It verifies that the development of Internet information channels has enriched the theories of information dissemination and consumer information behavior. Third, the study is more refined in terms of data and methodology, utilizing representative and timely microdata of Chinese residents to overcome the impact of sample area bias. Additionally, various methods such as instrumental variable techniques are employed for robustness and endogeneity analysis, aiming to address endogeneity issues related to variables related to internet usage. Fourth, through the analysis of heterogeneity among different occupational statuses and income levels, it is found that there are differences in the impact of internet usage on residents’ travel consumption before and after retirement. Specifically, compared to before retirement, residents are more likely to engage in travel consumption after retirement if they use the internet more frequently.

## Theoretical analysis

### Analysis of the impact of internet use on residents’ tourism consumption behavior

Consumer behavior theory and search theory are important theories in the field of marketing, helping us understand the decision-making process of consumers when purchasing products or services. Consumer behavior theory primarily studies the psychological, social, and cultural factors that influence consumers’ purchasing behavior during the buying process. Search theory, on the other hand, focuses more on the process of information search that consumers engage in before making a purchase, including information acquisition, processing, and decision-making. Analyze the impact mechanism of Internet usage on residents’ tourism consumption behavior based on consumer behavior theory and search theory.

The impact of internet use is everywhere, both at the macro level of our economic development and at the micro level of people’s lives. In residents’ daily lives, Internet use may affect their tourism consumption behaviour through a variety of channels, mainly including the following aspects. First, Internet use increases residents’ income and influences their tourism consumption behavior. Several studies have also confirmed that Internet use increases the income of the population through a variety of mechanisms, such as increased human capital, increased productivity and higher employment levels [21–23]. In economic terms, the economic base determines the superstructure. According to Maslow’s Hierarchy of Needs theory, especially for residents, an increase in income will lead to the pursuit of spiritual needs, such as outings, on top of satisfying physiological and security needs, etc. Secondly, Internet use has changed residents’ consumption perceptions and influenced their tourism consumption behaviour [24]. As a trading platform for tour operators and travellers, some information about transactions on the Internet and related reviews of tourism products can be saved. When residents browse the website for tourism information, the information on the existing tourism products and user reviews on the website are accessed by residents and become a reference for their travel decisions. In general, the idea of sensible and healthy consumption also spreads quickly through the Internet. Therefore, the Internet can help to stimulate the demand of the tourism market as well as help residents to break through the geographical restrictions in their consumption behaviour, leading to changes in their consumption attitudes and promoting their travel behaviour. Thus, we may obtain hypothesis 1.

Hypothesis 1: The Internet use has facilitated the tourist consumption behavior of the residents.

### Internet use and residents’ travel consumption behavior after retirement

According to sociological theory, retired individuals have more free time and energy, and therefore tend to use the internet more for information, socializing, entertainment, etc. The popularity and convenience of the internet make it easier for the retired population to access various travel information, thereby promoting their travel consumption behavior. In addition, the internet also provides retired individuals with more travel options and customized services, meeting their needs for different travel experiences.

In the current era of increasing ageing, tourism spending by the elderly is a new growth area for the future tourism economy. According to research data from CHARLS 2015, China’s elderly households already spend 9% of their money on travel. The theory of resident tourism consumption in relation to retirement can be traced back to Ando’s life-cycle consumption hypothesis, which suggests that residents’ consumption depends on income expectations and is smoothed over a lifetime, using the total resources they have as a constraint. Friedman started with the time of consumption and developed the persistent income hypothesis, which argues that persistent income is what sustains a stable level of consumption. The instantaneous change in income status after retirement and the more frequent use of the internet due to the presence of pension security and more leisure may have led to a change in resident travel consumption. Moreover, studies have shown that consumption of leisure and cultural entertainment is positively correlated with workers’ retirement [25]. Both the life-cycle consumption hypothesis and the persistent income hypothesis illustrate that people in retirement are more likely to consume, for example, cultural and recreational (i.e. travel) activities than those in pre-retirement due to their expected savings and stable sources of retirement income. According to the life-cycle consumption hypothesis, residents will smooth the distribution of consumption based on lifetime income. Compared to lower income groups, higher income groups are more likely to seek travel consumption to ease the strain of work due to their more frequent exposure to the internet. Combined with the persistent income hypothesis, there is a relative reduction in the expected income fluctuations of the higher income groups after retirement, which affects total tourism consumption. As they accumulate some income wealth, those with low incomes seek out travel and entertainment activities through frequent use of the internet in retirement. Thus, hypothesis 2 can be obtained.

Hypothesis 2: Retirement has a positive effect on the tourism consumption effect of internet use. Changes in income affect the distribution of travel spending among residents, with higher income groups being relatively less likely to spend on travel compared to lower income groups.

### Mediating effects of information access

In addition, the Internet can reduce the cost of travel, both in terms of time and material costs, facilitating access to travel information and promoting tourism consumption behavior. In general, before travelling, residents usually use various channels to obtain information about the destination or tourism products, such as travel agencies and traditional paper-based promotional materials. Compared to the traditional way of obtaining information, such as paper-based promotional materials, on the one hand, the Internet has further accelerated the speed of delivery of tourism-related information and broadened the scope of information coverage, prompting residents to choose the tourism products they are satisfied with in the shortest possible time; on the other hand, the Internet has broken through the limitations of time and space, making it possible for residents to search for tourism products anytime and anywhere, greatly reducing their search costs and promoting their tourism consumption behaviour.

Hypothesis 3: Internet use promotes residential tourism consumption behavior through access to information.

## Data sources and model setting

### Data sources

The data used in the article are from the China General Social Survey released by the National Survey Research Center at Renmin University of China in October 2020. The survey covered 28 provinces (autonomous regions and municipalities directly under the Central Government) in China, except for Tibet, Xinjiang and Hainan, and obtained 12,582 valid questionnaires. In this study, the sample was selected based on: (1) the explanatory variable (tourism consumption behaviour); (2) the core explanatory variable (internet use); and (3) the exclusion of those who refused to answer or answered “don’t know”. Based on the above processing, 9007 questionnaires were finally obtained and based on this, 9007 questionnaire data were finally used in the article.

### Variable selection

#### (1) Dependent variable

Tourism consumption behaviour. The CGSS questionnaire was used to identify travel consumption behavior based on the question “In the past year, how many nights did you stay away from home because you went on holiday or visited friends and relatives”. According to the respondents’ answers, those who answered “never” were considered as not having travel consumption behaviour and were assigned a value of 0. Those who answered “1–5 nights, 6–10 nights, 11–20 nights, etc.” were considered as having travel consumption behavior and were assigned a value of 1.

#### (2) Core independent variable

Internet use. The CGSS questionnaire was designed to identify the Internet use of the residents based on the question “In the past year, how did you use the Internet (including mobile phone access)”. Those who answered “never use” were assigned a value of 0; “rarely use” was assigned a value of 1; “sometimes use” was assigned a value of 2; “often use” was assigned a value of 3; and “very often use” was assigned a value of 4.

#### (3) Mediating variable

Access to information. According to the CGSS questionnaire, “Which is your main access to information”. Respondents who answered “Internet (including mobile phone access)” were assigned a value of 1, while those who answered “not Internet (including mobile phone access)” were assigned a value of 0.

#### (4) Control variable

With reference to existing studies, the variables of gender, age, education level, annual personal income, number of properties, health status and household economic status were selected as control variables, taking into account both individual and household characteristics of residents. Table 1 reports the variable definitions and descriptive statistics results.

**Table 1:** Variable definitions, assignments and their descriptive statistics.

Variable name	Meaning of variables	Average	SD
Residents’ tourism consumption behavior	0 = No; 1 = Yes	0.43	0.49
Internet use	0 = never use; 1 = rarely use; 2 = sometimes; 3 = often; 4 = very often	1.86	1.71
Access to information	0 = not from the Internet (mobile phone); 1 = from the Internet (mobile phone)	0.41	0.49
Gender	0 = female; 1 = male	0.512	0.500
Age	Continuous variable (years old)	50.67	16.26
Education level	1 = Primary school and below; 2 = Junior high school; 3 = Secondary or high school; 4 = College and above	2.27	1.11
Annual personal income	Continuous variables (taking logarithms)	9.94	1.30
Number of properties	Continuous variable (set)	0.73	0、66
Health status	1 = very unhealthy; 2 = relatively unhealthy; 3 = fair; 4 = relatively healthy; 5 = very healthy	3.49	1.07
Family financial situation	1 = far below the average level of income in society; 2 = below average; 3 = average; 4 = above average; 5 = well above average	2.58	0.75
Sample size	9007		

### Model setting

The Logit model can address the issue of non-normality in independent variables, and the probability values obtained fall within the range of [0,1], making it suitable for nonlinear situations. Residents’ travel consumption behavior is influenced by multiple factors, but the final outcome only has two possibilities, namely "yes" and "no", representing a binary variable within the range of [0,1]. Therefore, a binary logit model is used to analyze the impact of internet usage on residents’ travel consumption behavior. The Logit model was selected for empirical testing and the equation was as follows.

$$y^{*}=a+\beta \mathrm{internet}+\alpha \mathrm{information}+\sum_{n=1}\varepsilon Dni+\varsigma i$$ (1)

y*denotes the explanatory variable, which is tourism consumption behavior, internet denotes internet use, information denotes access to information, *Dni* stands for control variables such as individual characteristics and household characteristics,*α* stands for the constant term and *çi* stands for the error term.

In order to investigate the mechanism of access to information in the influence of Internet use on residents’ tourism consumption behaviour, the mediating effect model is used to empirically analyze the mechanism of action between Internet use, access to information and residents’ tourism consumption behaviour, drawing on the results of relevant studies. The following regression model was constructed.


$$Y_{i}=V_{1}+c_{i}T_{i}+b_{1i}X_{1i}+\varepsilon_{1i}$$ (2)

$$M_{i}=V_{2}+a_{i}T_{i}+b_{2i}X_{2i}+\varepsilon_{2i}$$ (3)

$$Y_{i}=V_{3}+c{'}_{i}T_{i}+d_{i}M_{i}+b_{3i}X_{3i}+\varepsilon_{3i}$$ (4)

Where *Y* denotes resident tourism consumption behaviour, *T* denotes the Internet use variable, *M* denotes the information access variable, *i* denotes a different resident, *X* is a control variable, *V* is a constant term, ε denotes a random disturbance term and *a*, *b*, *c* and *d* are regression coefficients.

## Analysis of the empirical results

### Baseline regression

This paper conducted a probit regression analysis of the effects of Internet use and access to information on residents’ tourism consumption behavior using Stata 15 software. Table 2 reports the results of the empirical tests. Before conducting the empirical tests, the explanatory variables were first tested for multicollinearity and the results showed a maximum VIF value of 2.28, a minimum VIF value of 1.05 and a mean VIF value of 1.53, with the maximum VIF value being significantly less than 10, indicating that no serious problem of multicollinearity exists between the selected explanatory variables.

**Table 2:** Regression results of the effect of Internet use on residents’ tourism consumption behavior.

	Logit	marginal effect	LPM	LDM
Internet use	0.222***	0.054***	0.051***	0.245***
	(0.019)	(0.005)	(0.004)	(0.020)
Gender	-0.242***	-0.059***	-0.049***	-0.223***
	(0.055)	(0.013)	(0.011)	(0.049)
Age	-0.008***	-0.002***	-0.002***	-0.008***
	(0.002)	(0.000)	(0.000)	(0.002)
Education level	0.235***	0.057***	0.053***	0.254***
	(0.032)	(0.008)	(0.007)	(0.028)
Annual personal income	0.115***	0.028***	0.02***	0.089***
	(0.031)	(0.007)	(0.005)	(0.026)
Number of properties	0.020	0.005	0.004	0.017
	(0.039)	(0.010)	(0.008)	(0.038)
Health status	0.020	0.005	0.004	0.016
	(0.033)	(0.008)	(0.007)	(0.025)
Family financial situation	0.181***	0.044***	0.037***	0.167***
	(0.032)	(0.008)	(0.007)	(0.035)
Provincial fixed effects	Yes	Yes	Yes	Yes

Note

*, ** and *** indicate significant at the 10%, 5% and 1% levels respectively.

Table 2 reports the regression results of Internet use on resident tourism consumption. Both Logit and its marginal effects, as well as the estimation results of LPM and LDM, show that the coefficient of the effect of Internet use on residential tourism consumption behavior is significantly positive, suggesting that Internet use is favourable to promote residential tourism consumption behavior, which verifies hypothesis 1. The reason for this is the fact that the Internet use has accelerated and widened the speed and scope of the dissemination of tourism-related information. This enables residents to access information about tourism resources and other related information from the Internet in a more convenient and quicker way, and to purchase tourism products and plan their travel routes whenever and wherever they want, thus reducing the ’spatial distance’ between residents and tourism resources to a certain extent. It can be concluded that the Internet use has stimulated the residents’ tourism demand potential and desire to travel. Internet technology has removed the constraints of time and space in the tourism market, expanding the choices available to tourists and making travel consumption more convenient. This has increased the overall utility level of tourists.

The impact of the control variables on residents’ tourism consumption behaviour varies. The coefficients of the effects of gender and age on residents’ travel consumption behavior are negative and both pass the test at the 1% level of significance, which indicates that the older the resident is, the less likely it is that travel consumption behavior will occur. Moreover, women are more likely to engage in travel spending behavior than men. This may be due to the traditional Chinese model of "male lord outside homemaking women”. Men are too busy with their careers to travel, while women are more inclined to spend money on travel for enjoyment or relaxation. This indicates that female residents have more "decision-making power" in tourism consumption. In addition, residents’ education level, annual income and household financial status contribute to their travel behavior. It indicates that the higher the education level of residents, the more likely their tourism consumption behavior will occur; the higher the income level of residents, the higher the possibility of tourism consumption occurring; and the better the financial situation of residents’ households, the more likely their tourism consumption behavior will be promoted. The effect of the number of properties and health status of residents on their travel consumption behaviour was small and did not pass the significance test.

At the same time, the paper reveals through Logit and LPM relative importance statistics that Internet use makes the greatest contribution to residents’ travel consumption behavior, followed by educational attainment and annual personal income, then age. However, the contribution coefficient for age is less than 0.02 and the contribution coefficients for gender, number of properties, health status and household economic status are relatively low at less than 0.01. From this, it can be inferred that the impact of internet use on residents’ travel consumption behavior is more likely to be constrained by the moderation of educational attainment and annual personal income. To this point, this is subject to verification by the following heterogeneity analysis.

### Heterogeneity analysis

In China, the retirement age is generally 60 years for residents. Generally speaking, retirement allows residents to spend more free time for enjoyment of life or to engage in consumption behavior for spiritual enjoyment (e.g. travel). As can be seen from Table 2, the contribution of annual personal income to residents’ travel consumption behavior is 0.021 and 0.026 respectively, judging from the significance of Logit and LPM. As a result, this paper classifies residents as 1 if they are over 60 years old and 0 if they are under 60 years old; and divides annual personal income into low-income and high-income groups according to median and conducts heterogeneity analysis as in Table 3. Both before and after retirement, Internet use boosts residents tourism consumption behavior, with the latter having an impact coefficient approximately 0.102 higher than that of the former. A p-value of 0.0147, significant at the 5% level, was found when the two were tested for conjunction. This suggests that internet use is more likely to drive residents tourism consumption behavior when they retire than before retirement. Columns 4 and 5 of Table 3 show that the interaction term between high income and Internet use are both positive, which indicates that people with high income are more likely to make residential tourism purchases when using the Internet compared to those with low income. By comparing the pre- and post-retirement groups (see columns 6 and 7 of Table 3), the interaction term between high income and Internet use is negative in the post-retirement group columns and positive in the pre-retirement group columns. This suggests that as residents retire, the more frequently those with higher incomes use the Internet, the less likely residents are to engage in tourism consumption behaviour. It may be due to the fact that those with higher incomes may be less inclined to travel after retirement and more likely to choose to spend on tourism before retirement to ease the tedium of a busy work schedule. On balance, the more frequently the retired residents use the Internet compared to the pre-retirement period, the more likely they are to spend on travel. Compared to the low-income group, the more frequently the high-income group uses the Internet after retirement, the more likely residents are not necessarily to increase their travel spending, which verifies hypothesis 2. Through heterogeneity analysis, it was found that income level is one of the important economic factors affecting residents’ consumption. An increase in income level can drive the growth of Chinese residents’ tourism consumption.

**Table 3:** Results of heterogeneity analysis.

	Pre-retirement	Post-retirement	Logit	LDM	Pre-retirement	Post-retirement
Internet use	0.190***	0.302***	0.206***	0.184***	0.223***	0.441***
	(0.024)	(0.039)	(0.022)	(0.023)	(0.027)	(0.054)
High income			0.211**	0.133	0.406***	0.099
			(0.105)	(0.089)	(0.144)	(0.126)
High Income #Internet Use			0.045	0.085***	0.008	-0.157***
			(0.031)	(0.030)	(0.041)	(0.060)
Control variable	YES	YES	YES	YES	YES	YES
Provincial fixed effect	YES	YES	YES	YES	YES	YES
Sample size	9007		9007	9007	6205	2802

Note

*, ** and *** indicate significant at the 10%, 5% and 1% levels respectively, with robust standard errors in parentheses. Columns 2, 3, 6 and 7 are logit estimations.

### Robustness tests

#### (1) Variable substitution

The results of the replacement model regressions are reported in Table 4. The explanatory variables in this paper are categorical variables of type 0 versus 1. To explore the robustness of the findings, the paper continues to use a logit model to analyze the relationship between internet use, access to information and residents tourism consumption behavior. It shows that Internet use passes the significance test at the 1% level, which is consistent with the findings in Table 2, suggesting that the research findings that Internet use is conducive to promoting residents tourism consumption behaviour are robust.

**Table 4:** Regression results of the replacement model.

	Coefficient	Standard error
Internet use	0.233***	0.019
Control variable	Controlled	Controlled
Sample size	9007	

Note

*, ** and *** indicate significant at the 10%, 5% and 1% levels respectively.

#### (2) Endogenous test

The results of the previous study suggest that Internet use can facilitate the occurrence of residents tourism consumption behavior. However, there may be reverse causality, whereby residents use the Internet in order to travel and consume away from home, and thus endogeneity problems arise, leading to biased findings. In addition, there is the issue of omitted variables. Certain omitted variables may affect or even determine internet usage patterns, while they may also be related to tourism expenditure. Based on this, in order to reduce possible endogenous problems that may affect the findings of the paper, an instrumental variables approach was adopted to accurately estimate the impact of Internet use on residents tourism consumption behavior in order to prove the reliability of the study findings.

To address this issue, the “number of personal Internet devices owned” was chosen as the instrumental variable for whether or not residents use the Internet. Theoretically, instrumental variables must meet the characteristics of relevance and exogeneity. In terms of relevance, the "number of personal Internet devices owned" is a measure of the number of personal Internet devices. The greater the number of devices that residents have access to the Internet, the greater the possibility that they will be able to use it. From an exogenous perspective, it is difficult for the number of devices on which residents have access to the Internet to directly influence their travel consumption behavior. Logically, therefore, the “number of personal Internet devices owned” satisfies the correlation and exogeneity characteristics. Table 5 shows the results of the endogeneity test. In column I, the F value is much greater than 16.38, indicating that the research does not have a weak instrumental variable problem. The p-value for the overidentification test was 0.2242, indicating that the original hypothesis of exogenous instrumental variables could not be rejected. Therefore, the “number of personal Internet devices owned” is appropriate as an instrumental variable for Internet use. The regression results in Column I and Column II indicate that Internet use both pass the significance test at the 1% level, which also suggests the robustness of the findings that Internet use helps promote residents tourism consumption behavior. In conclusion, considering endogeneity issues, regardless of the model used, internet usage significantly increases residents’ expenditure on tourism consumption. Therefore, hypothesis 1 is once again validated.

**Table 5:** Endogenous test.

Variable	Coefficient (standard error)	Coefficient (standard error)
	2SLS	LIML
Internet use	0.087***(0.026)	0.087***(0.025)
Control variable	Controlled	Controlled
Over-identification test p-value	0.2242	
Wald F-statistic value	1649.47	
Sample size	9007	9007

Note

*, ** and *** indicate significant at the 10%, 5% and 1% levels respectively.

### Mechanism of action test

As analyzed earlier, internet use may facilitate residents’ access to travel information, thereby having an impact on their tourism consumption behavior. Based on this, a mediating effects model was adopted to test the relationship between access to information in Internet use and residents tourism consumption behavior, drawing on existing research findings. Table 6 reports the results of the tests for mediating effects.

**Table 6:** Results of the intermediary effect mechanism test.

	Residents tourism consumption behavior	Access to information	Residents tourism consumption behavior
	Logit	Logit	Logit
	Coefficient (standard error)	Coefficient (standard error)	Coefficient (standard error)
Internet use	0.145*** (0.011)	0.716*** (0.018)	0.129*** (0.014)
Access to information	---	---	0.127*** (0.045)
Control variable	Controlled	Controlled	Controlled
Sample size	9007		

Note

*, ** and *** indicate significant at the 10%, 5% and 1% levels respectively.

In Table 6, access to information plays a partially mediating role between Internet use and residents tourism consumption behavior. Specifically, compared to column Ⅰ, the coefficient of the effect of Internet use on residents tourism consumption behavior decreases in column Ⅲ. Access to information all passed the positive significance test at the 1% level, indicating that it plays a partially mediating, not fully mediating, role in the relationship between Internet use and residents tourism consumption behavior, according to the classical test for mediating effects. This suggests that Internet use can broaden residents’ access to information and promote tourism consumption behavior, which verifies hypothesis 3.

The popularization and application of the Internet have provided residents with a wider channel for obtaining information, especially in the field of tourism. Through the Internet, residents can easily access travel information about their destination, including introductions to attractions, transportation routes, accommodation options, food recommendations, etc., helping them better plan their travel itineraries. In addition, the Internet also offers online booking services, allowing residents to conveniently book flights, hotels, tickets, etc., saving time and effort. Through the Internet, residents can participate in various travel-related social platforms and forums, share experiences with other travelers, exchange opinions, and obtain more practical travel advice. This information sharing and interaction promote the occurrence of travel consumption behavior, increasing people’s interest and enthusiasm for travel. At the same time, the Internet also provides residents with more choices and opportunities for comparison, helping them find travel products and services with better value for money, thereby promoting the development and prosperity of the tourism market.

## Conclusion and implications

Compared with existing studies, this article, for the first time, conducts a heterogeneous analysis of the impact of internet use on residents’ tourism consumption from the perspectives of work status differentiation, income stratification, and other aspects. Given that residents’ tourism consumption is time-constrained, this article selects employment status and occupation type in heterogeneity analysis based on micro-database data to indirectly reflect residents’ leisure status, and demonstrates the significant role of leisure time in residents’ tourism consumption.

Combining consumer behavior theory, search theory, and sociology theory, Based on 9007 China General Social Survey (CGSS) data in 2017, this paper explores the impact of internet use and access to information on residents tourism consumption behavior. The descriptive statistics of the data revealed that 43% of the sample residents had spent money on travel, and the mean value of Internet use of the sample residents was 1.86. According to further analysis using Logit and mediating effects models, it is concluded that Internet use can promote residents tourism consumption behavior; access to information can also have an impact on residents tourism consumption behavior to a certain extent. The results of the mechanism of action show that access to information plays a partially mediating role between Internet use and residents tourism consumption behavior, suggesting that Internet use can promote residents tourism consumption behavior by expanding information channels. In addition, retirement positively affects resident tourism consumption behavior, positively moderating the effect of internet use on resident tourism consumption behavior, while rising incomes reduce the effect of retirement on resident tourism consumption. The impact of variables such as the age of residents on their travel consumption behavior varies. Robustness tests using the replacement estimation model and the instrumental variables approach concluded that the findings of the study on Internet use to promote residents tourism consumption behavior remain robust. Based on this, the following can be drawn.

Firstly, Internet is a medium for information transfer and an important basis for the implementation of online promotion for tourism resources. For tourist attractions and rural areas with poor internet infrastructure, funding should be continuously increased to stimulate the tourism consumption potential of residents, especially rural residents, by improving internet infrastructure. In addition, it should promote network speed and tariff reduction to improve the accessibility of network resources to residents and broaden their access to tourism-related resources. Secondly, tourism enterprises or relevant tourist attractions should, while enriching offline tourism products, continuously strengthen online publicity through various means such as prize competitions and travelogues competitions, make use of the characteristics of fast and wide dissemination of the Internet to expand publicity coverage, enrich Internet network tourism products and increase residents’ perception of the image of tourism products. At the same time, there is a need to develop personalized products for different types or age groups of tourism consumers in an appropriate manner. Finally, it is important to optimize the online environment for tourism resources, enhance residents’ trust, protect their travel rights and promote their courage to access tourism-related information via the Internet.

## Limitations and future research directions

With the rapid development of the Internet and the further integration of Internet technology with residents’ consumption, this research still has many shortcomings, and future exploration of related content needs to be further deepened. Limitations include: firstly, constrained by macro databases, the selection of variables may not be targeted enough, overlooking the impact of external factors such as air pollution on residents’ travel consumption; secondly, data may become outdated, leading to incomplete consideration of issues. Therefore, in future research, firstly, consider the impact of the external environment on individual behavior; secondly, on the basis of using macro panel data, match it with micro survey data to make the research more comprehensive and thorough.

## Supporting information

S1 Data (XLSX)

## References

[1] Mbaiwa J.E., The socio-economic and environmental impacts of tourism development on the Okavango Delta, north-western Botswana[J]. Journal of arid environments, 2003, 54(2): 447–467. 10.1006/jare.2002.1101.

[2] Rosselló-Nadal J., How to evaluate the effects of climate change on tourism[J]. Tourism Management, 2014, 42: 334–340. 10.1016/j.tourman.2013.11.006.

[3] Zhang A., Zhong L., Xu Y., Wang H., and Dang L., Tourists’ perception of haze pollution and the potential impacts on travel: Reshaping the features of tourism seasonality in Beijing, China[J]. Sustainability, 2015, 7(3): 2397–2414. 10.3390/su7032397.

[4] Chan C.S., and Yuan J., Changing travel behaviour of high-speed rail passengers in China[J]. Asia Pacific Journal of Tourism Research, 2017, 22(12): 1221–1237. 10.1080/10941665.2017.1391303.

[5] Algassim A.A., and Abuelhassan A.E., The Effect of COVID-19 on potential tourist’s consumption behavior: Evidence from GCC countries[J]. Journal of Association of Arab Universities for Tourism and Hospitality, 2021, 20(1): 129–144. doi: 10.21608/JAAUTH.2021.55964.1108

[6] Li X., Li X.R., and Hudson S., The application of generational theory to tourism consumer behavior: An American perspective[J]. Tourism Management, 2013, 37: 147–164. 10.1016/j.tourman.2013.01.015.

[7] Budeanu A., Sustainable tourist behaviour–a discussion of opportunities for change[J]. International journal of consumer studies, 2007, 31(5): 499–508. 10.1111/j.1470-6431.2007.00606.x.

[8] Yoon A., Jeong D., and Chon J., The impact of the risk perception of ocean microplastics on tourists’ pro-environmental behavior intention[J]. Science of the Total Environment, 2021, 774: 144782. 10.1016/j.scitotenv.2020.144782.

[9] Yi S., Day J., and Cai L.A., Exploring tourist perceived value: An investigation of Asian cruise tourists’ travel experience[J]. Journal of Quality Assurance in Hospitality & Tourism, 2014, 15(1): 63–77. 10.1080/1528008X.2014.855530.

[10] Tang C., Han Y., and Ng P., Green consumption intention and behavior of tourists in urban and rural destinations[J]. Journal of Environmental Planning and Management, 2023, 66(10): 2126–2150. 10.1080/09640568.2022.2061927.

[11] Nguyen C.P., Thanh S.D., and Nguyen B., Economic uncertainty and tourism consumption[J]. Tourism Economics, 2022, 28(4): 920–941. 10.1177/1354816620981519.

[12] Santos V., Ramos P., Sousa B., Almeida N., and Valeri M., Factors influencing touristic consumer behaviour[J]. Journal of Organizational Change Management, 2022, 35(3): 409–429. 10.1108/JOCM-02-2021-0032.

[13] Ataul K.P., Roslizawati C.A., and Nik A.A., Investigating tourists’ intention toward green hotels in Malaysia: a direction on tourist sustainable consumption[J].Environmental Science and Pollution Research.2023,30(13):38500–38511. doi: 10.1007/s11356-022-24946-x 36580253

[14] Xiang Z., Magnini V.P., and Fesenmaier D.R., Information technology and consumer behavior in travel and tourism: Insights from travel planning using the internet[J]. Journal of retailing and consumer services, 2015, 22: 244–249. 10.1016/j.jretconser.2014.08.005.

[15] Aldebert B., Dang R.J., and Longhi C., Innovation in the tourism industry: The case of Tourism@[J]. Tourism management, 2011, 32(5): 1204–1213. 10.1016/j.tourman.2010.08.010.

[16] Vacation E., Analysis of ICT usage patterns, benefits and barriers in tourism SMEs in the Middle Eastern countries: The case of Dubai in UAE[J]. Journal of Engineering, 2017,23,(3):248–263. 10.1177/1356766716654515.

[17] Tran M.T., Jeeva A.S., and Pourabedin Z., Social network analysis in tourism services distribution channels[J]. Tourism Management Perspectives, 2016, 18: 59–67. 10.1016/j.tmp.2016.01.003.

[18] Benckendorff P.J., and Black N.L., Destination marketing on the Internet: A case study of Australian Regional Tourism Authorities[J]. Journal of Tourism Studies, 2000, 11(1): 11–21. http://espace.library.uq.edu.au/view/UQ:240532.

[19] Senyao S., and Ha S., How social media influences resident participation in rural tourism development: a case study of Tunda in Tibet[J]. Journal of Tourism and Cultural Change, 2020(1):1–20. doi: 10.1080/14766825.2020.1849244

[20] Xu X., and Pratt S., Social media influencers as endorsers to promote travel destinations: an application of self-congruence theory to the Chinese Generation Y[J]. Journal of travel & tourism marketing, 2018, 35(7): 958–972. 10.1080/10548408.2018.1468851.

[21] Moretti E., Human capital externalities in cities[M]//Handbook of regional and urban economics. Elsevier, 2004, 4: 2243–2291. 10.3386/w9641.

[22] DiMaggio P., and Bonikowski B., Make money surfing the web? The impact of Internet use on the earnings of US workers[J]. American Sociological Review, 2008, 73(2): 227–250. 10.1177/000312240807300203.

[23] Galperin H., and Fernanda V. M., Connected for development? Theory and evidence about the impact of internet technologies on poverty alleviation[J]. Development Policy Review, 2017, 35(3): 315–336. 10.1111/dpr.12210.

[24] Cantallops A.S., and Salvi F., New consumer behavior: A review of research on eWOM and hotels[J]. International Journal of Hospitality Management, 2014, 36: 41–51. 10.1016/j.ijhm.2013.08.007.

[25] Becchetti L., Giachin Ricca E., and Pelloni A., The relationship between social leisure and life satisfaction: Causality and policy implications[J]. Social indicators research, 2012, 108(3): 453–490. 10.1007/s11205-011-9887-5.

